# Integrating virtual patients into undergraduate health professions curricula: a framework synthesis of stakeholders’ opinions based on a systematic literature review

**DOI:** 10.1186/s12909-024-05719-1

**Published:** 2024-07-05

**Authors:** Joanna Fąferek, Pierre-Louis Cariou, Inga Hege, Anja Mayer, Luc Morin, Daloha Rodriguez-Molina, Bernardo Sousa-Pinto, Andrzej A. Kononowicz

**Affiliations:** 1 Center for Innovative Medical Education, Jagiellonian University Medical College, Medyczna 7, Krakow, 30-688 Poland; 2 Faculty of Medicine, Paris Saclay University, Le Kremlin-Bicetre, 94270 France; 3 Paracelsus Medical University, Prof.-Ernst-Nathan-Str. 1, 90419 Nürnberg, Germany; 4 Medical Education Sciences, University of Augsburg, 86159 Augsburg, Germany; 5 Institute and Clinic for Occupational, Social and Environmental Medicine, LMU University Hospital, 80336 Munich, Germany; 6 Department of Community Medicine, Information and Health Decision Sciences, Faculty of Medicine, University of Porto, Porto, Portugal; 7 Department of Bioinformatics and Telemedicine, Jagiellonian University Medical College, Medyczna 7, Krakow, 30-688 Poland

**Keywords:** Virtual patients, Curriculum development, Systematic review, Framework synthesis

## Abstract

**Background:**

Virtual patients (VPs) are widely used in health professions education. When they are well integrated into curricula, they are considered to be more effective than loosely coupled add-ons. However, it is unclear what constitutes their successful integration. The aim of this study was to identify and synthesise the themes found in the literature that stakeholders perceive as important for successful implementation of VPs in curricula.

**Methods:**

We searched five databases from 2000 to September 25, 2023. We included qualitative, quantitative, mixed-methods and descriptive case studies that defined, identified, explored, or evaluated a set of factors that, in the perception of students, teachers, course directors and researchers, were crucial for VP implementation. We excluded effectiveness studies that did not consider implementation characteristics, and studies that focused on VP design factors. We included English-language full-text reports and excluded conference abstracts, short opinion papers and editorials. Synthesis of results was performed using the framework synthesis method with Kern’s six-step model as the initial framework. We appraised the quality of the studies using the QuADS tool.

**Results:**

Our search yielded a total of 4808 items, from which 21 studies met the inclusion criteria. We identified 14 themes that formed an integration framework. The themes were: goal in the curriculum; phase of the curriculum when to implement VPs; effective use of resources; VP alignment with curricular learning objectives; prioritisation of use; relation to other learning modalities; learning activities around VPs; time allocation; group setting; presence mode; VPs orientation for students and faculty; technical infrastructure; quality assurance, maintenance, and sustainability; assessment of VP learning outcomes and learning analytics. We investigated the occurrence of themes across studies to demonstrate the relevance of the framework. The quality of the studies did not influence the coverage of the themes.

**Conclusions:**

The resulting framework can be used to structure plans and discussions around implementation of VPs in curricula. It has already been used to organise the curriculum implementation guidelines of a European project. We expect it will direct further research to deepen our knowledge on individual integration themes.

**Supplementary Information:**

The online version contains supplementary material available at 10.1186/s12909-024-05719-1.

## Introduction

Virtual patients (VPs) are defined as interactive computer simulations of real-life clinical scenarios for the purpose of health professions training, education, or assessment [1]. Several systematic reviews have demonstrated that learning using VPs is associated with educational gains when compared to no intervention and is non-inferior to traditional, non-computer-aided, educational methods [2–4]. This conclusion holds true across several health professions, including medicine [3, 5], nursing [6] and pharmacy [7]. The strength of VPs in health professions education lies in fostering clinical reasoning [4, 6, 8] and related communication skills [5, 7, 9]. At the same time, the research syntheses report high heterogeneity of obtained results [2, 4]. Despite suggestions in the literature that VPs that are well integrated into curricula are more effective than loosely coupled add-ons [5, 10, 11], there is no clarity on what constitutes successful integration. Consequently, the next important step in the research agenda around VPs is to investigate strategies for effectively implementing VPs into curricula [9, 12, 13].

In the context of healthcare innovation, implementation is the process of uptaking a new finding, policy or technology in the routine practice of health services [14–16]. In many organisations, innovations are rolled out intuitively, which at times ends in failure even though the new tool has previously shown good results in laboratory settings [17]. A large review of over 500 implementation studies showed that better-implemented health promotion programs yield 2–3 times larger mean effect sizes than poorly implemented ones [18]. Underestimation of the importance and difficulty of implementation processes is costly and may lead to unjustified attribution of failure to the new product, while the actual problem is inadequate methods for integration of the innovation into practice [15].

The need for research into different ways of integrating computer technology into medical schools was recognised by Friedman as early as 1994 [19]. However, studies of the factors and processes of technology implementation in medical curricula have long been scarce [12]. While the terminology varies across studies, we will use the terms *introduction, integration, incorporation*, and *implementation* of VPs into curricula interchangeably. Technology *adoption* is the decision to use a new technology in a curriculum, and we view it as the first phase of implementation. In an early guide to the integration of VPs into curricula, Huwendiek et al. recommended, based on their experience, the consideration of four aspects relevant to successful implementation: blending face-to-face learning with on-line VP sessions; designing collaborative learning around VPs; allowing students flexibility in deciding when/where/how to learn with VPs; and constructively aligning learning objectives with suitable VPs and matched assessment [20]. In a narrative review of VPs in medical curricula, Cendan and Lok identified a few practices which are recommended for the use of VPs in curricula: filling gaps in clinical experience with standardised and safe practice, replacing paper cases with interactive models showing variations in clinical presentations, and providing individualised feedback based on objective observation of student activities. These authors also highlighted cost as a significant barrier to the implementation process [21]. Ellaway and Davies proposed a theoretical construct based on Activity Theory to relate VPs to their use and to link to other educational interventions in curricula [22]. However, a systematic synthesis of the literature on the identified integration factors and steps relevant to VP implementation is lacking.

The context of this study was a European project called iCoViP (International Collection of Virtual Patients; https://icovip.eu*)*, which involved project partners from France, Germany, Poland, Portugal, and Spain and succeeded in creating a collection of 200 open-access VPs available in 6 languages to support clinical reasoning education [23]. Such a collection would benefit from being accompanied by integration guidelines to inform potential users on how to implement the collection into their curricula. However, guidelines require frameworks to structure the recommendations. Existing integration frameworks are limited in scope for a specific group of health professions, were created mostly for evaluation rather than guidance, or are theoretical or opinion-based, without an empirical foundation [24–26].

Inspired by the methodological development of qualitative literature synthesis [27], we decided to build a mosaic of the available studies in order to identify and describe what stakeholders believe is important when planning the integration of VPs into health professions curricula. The curriculum stakeholders in our review included students, teachers, curriculum planners, and researchers in health professions education. We aimed to develop a framework that would configure existing research on curriculum implementations, structure future practice guidelines, and inform research agendas in order to strengthen the evidence behind the recommendations.

Therefore, the research aim of this study was to identify and synthesise themes across the literature that, in stakeholders’ opinions, are important for the successful implementation of VPs in health professions curricula.

## Methods

This systematic review is reported in accordance with the Preferred Reporting Items for Systematic Reviews and Meta-Analyses (PRISMA) framework [28].

### Eligibility criteria

We selected studies whose main objective was to define, identify, explore, or evaluate a set of factors that, in the view of the authors or study participants, contribute to the successful implementation of VPs in curricula. Table 1 summarises the inclusion and exclusion criteria.

**Table 1:** Key inclusion and exclusion criteria of the review

Criteria	Inclusion	Exclusion
Population	• Stakeholders involved in the implementation or use of VPs in undergraduate programs, such as: o students, o academic teachers, o clerkship directors, or o researchers in health professions curricula.	• Curricula addressed not to students but to professionals or patients. • VPs for use outside of education. • Curricula for students of traditional, alternative and complementary medicine. • Postgraduate curricula.
Intervention	• Interactive VP scenarios with a patient history that unfolds over time. • Primary target outcome of the VP is clinical reasoning or related, e.g., communication skills.	• Scenarios that require non-standard equipment or are human-controlled. • The primary outcome is not related to the diagnostic or management process of the patient. • Extracurricular events outside the regular study program.
Outcome	• The outcome of the study is a set of factors relevant to the integration of VPs into health professions curricula. • The study provides models for integrating VPs or tools for evaluating integration. • Papers that aim to investigate why VPs did/did not work in curricula.	• Papers that aimed to evaluate whether VPs were effective as a teaching method but do not go into detail about how they were integrated into curricula.
Study Design	• Research studies (qualitative and quantitative, mixed- methods, descriptive).	• Opinion papers and editorials. • Studies carried out in laboratory settings or in the context of extracurricular subjects. • Studies on the effectiveness or efficacy of VP design, student satisfaction or motivation.

The curricula in which VPs were included targeted undergraduate health professions students, such as human medicine, dentistry, nursing, or pharmacy programs. We were interested in the perspectives of all possible stakeholders engaged in planning or directly affected by undergraduate health professions curricula, such as students, teachers, curriculum planners, course directors, and health professions education researchers. We excluded postgraduate and continuing medical education curricula, faculty development courses not specifically designed to prepare a faculty to teach an undergraduate curriculum with VPs, courses for patients, as well as education at secondary school level and below. Also excluded were alternative and complementary medicine programs and programs in which students do not interact with human patients, such as veterinary medicine.

Similar to the previous systematic review [4], we excluded from the review VP simulations that required non-standard computer equipment (like virtual reality headsets) and those in which the VP was merely a static case vignette without interaction or the VP was simulated by a human (e.g., a teacher answering emails from students as a virtual patient). We included studies in which VPs were presented in the context of health professions curricula; we excluded studies in which VPs were used as extracurricular activities (e.g., one-time learning opportunities, such as conference workshops) or merely as part of laboratory experimentation.

We included all studies that presented original research, and we excluded editorials and opinion papers. Systematic reviews were included in the first stage so we could manually search for references in order to detect relevant studies that had potentially been omitted. We included studies that aimed to comprehensively identify or evaluate external contextual factors relevant for the integration of VPs into curricula or that examined activities around VPs and the organisational, curricular and accreditation context (the *constructed* and *framed* layers of activities in Ellaway & Davies’ model [22]). As the goal was to investigate integration strategies, we excluded VP design studies that looked into techniques for authoring VPs or researched technical or pedagogical mechanisms encoded in VPs that could not be easily altered (i.e., *encoded* layer of VP activities [22]). As we looked into studies that comprehensively investigated a set of integration factors that are important in the implementation process, we excluded studies that focus on program effectiveness (i.e., whether or not a VP integration worked) but do not describe in detail how the VPs were integrated into curricula or investigate what integration factors contributed to the implementation process. We also excluded studies that focused on a single integration factor as our goal was to explore the broad perspective of stakeholders’ opinions on what factors matter in integration of VPs into curricula.

We only included studies published in English as we aimed to qualitatively analyse the stakeholders’ opinions in depth and did not want to rely on translations. We chose the year 2000 as the starting point for inclusion. We recognise that VPs were used before this date but also acknowledge the significant shift in infrastructure from offline technologies to the current web-based platforms, user-friendly graphical web browsers, and broadband internet, all of which appeared around the turn of the millennium. Additionally, VP literature before 2000 was mainly focused on demonstrating technology rather than integrating these tools into curricula [12, 19].

### Information sources and search

We systematically searched the following five bibliographic databases: MEDLINE (via PubMed), EMBASE (via Elsevier), Educational Resource Information Center (ERIC) (via EBSCO), CINAHL Complete (via EBSCO), Web of Science (via Clarivate). The search strategies are presented in Supplementary Material S1. We launched the first query on March 8, 2022, and the last update was carried out on September 25, 2023. The search results were imported into the Rayyan on-line software [29]. Duplicate items were removed. Each abstract was screened by at least two reviewers working independently. In the case of disagreement between reviewers, we included the abstract for full text analysis. Next, we downloaded the full text of the included abstracts, and pairs of reviewers analysed the content in order to determine whether they met the inclusion criteria. In the case of disagreement, a third reviewer was consulted to arbitrate the decision.

### Data extraction and analysis

Reviewers working independently extracted relevant characteristics of the included studies to an online spreadsheet. We extracted such features as the country in which the study was conducted, the study approach, the data collection method, the year of implementation in the curriculum, the medical topic of the VPs, the type and number of participants, the number of included VPs, the type of VP software, and the provenance of the cases (e.g., self-developed, part of a commercial database or open access repository).

The qualitative synthesis followed the five steps of the framework synthesis method [27, pp. 188–190]. In the familiarisation phase (step 1), the authors who were involved previously in the screening and data extraction process read the full text versions of the included studies to identify text segments containing opinions on how VPs should be implemented into curricula.

Next, after a working group discussion, we selected David Kern’s six-step curriculum development [30] for the pragmatic initial frame (step 2). Even though it is not a VP integration framework in itself, we regarded it as a “best fit” to configure a broad range of integration factors spanning the whole process of curriculum development. David Kern’s model is often used for curriculum design and reform and has also been applied in the design of e-learning curricula [31]. Through a series of asynchronous rounds of comments, on-line meetings and one face-to-face workshop that involved a group of stakeholders from the iCoViP project, we iteratively clustered the recommendations into the themes that emerged. Each theme was subsumed to one of Kern’s six-steps in the initial framework. Next, we formulated definitions of the themes.

In the indexing phase (step 3), two authors (JF and AK) systematically coded the results and discussion sections of all the included empirical studies, line-by-line, using the developed themes as a coding frame. Text segments grouped into individual themes were comparatively analysed for consistency and to identify individual topics within themes. Coding was performed using MaxQDA software for qualitative analysis (MaxQDA, version 22.5 [32]). Disagreements were discussed and resolved by consensus, leading to iterative refinements of the coding frame, clarifications of definitions, and re-coding until a final framework was established.

Subsequently, the studies were charted (step 4) into tables in order to compare their characteristics. Similar papers were clustered based on study design to facilitate closer comparisons. A quality appraisal of the included studies was then performed using a standardised tool. Finally, a visual representation of the framework was designed and discussed among the research team, allowing for critical reflection on the consistency of the themes.

In the concluding step (step 5), in order to ensure the completeness and representativeness of the framework for the analysed body of literature, we mapped the themes from the developed framework to the studies in which they were found, and we analysed how individual themes corresponded to the conceptual and implementation evaluation models identified during the review. We looked for patterns and attempted to interpret them. We also looked for inconsistencies and tensions in the studies to identify potential areas for future research.

### Quality appraisal of the included studies

To appraise the quality of the included studies, we selected the QuADS (Quality Assessment with Diverse Studies) tool [33], which is suitable for assessing the quality of studies with diverse designs, including mixed- or multi-method studies. This tool consists of 13 items on a four-point scale (0: not reported; 1: reported but inadequate; 2: reported and partially adequate; 3: sufficiently reported). QuADS has previously been successfully used in synthesis of studies in the field of health professions education [34] and technology-enhanced learning environments [35]. The included qualitative studies, quantitative surveys, and mixed-methods interview studies were independently assessed by two reviewers (JF, AK). The results were then compared; if differences arose, the justifications were discussed and a final judgement was reached by consensus. Following the approach taken by Goagoses et al. [35], we divided the studies into three groups, depending on the summary quality score: weak (≤ 49% of QuADS points); medium (50–69%) and high (≥ 70%) study quality.

## Results

### Characteristics of the included studies

**Fig. 1:**
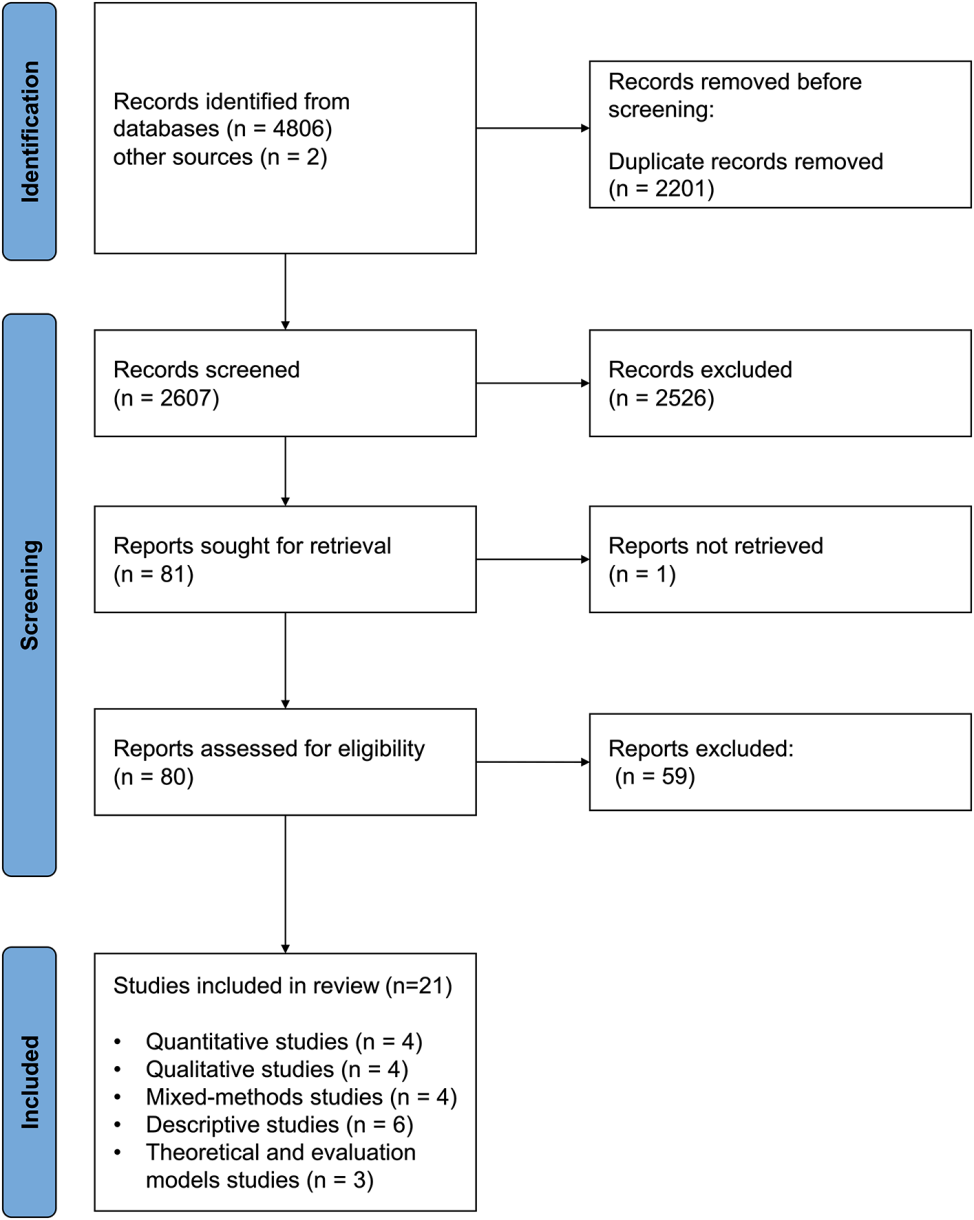
PRISMA flowchart of the study selection process

The selection process for the included studies is presented in Fig. 1.

Our search returned a total of 4808 items. We excluded duplicate records (*n* = 2201), abstracts not meeting the inclusion criteria (*n* = 2526), and complete reports (*n* = 59) after full text analysis. In the end, 21 studies met our inclusion criteria.

### Types of included studies

In the analysis of the 21 included studies, 18 were classified as empirical studies, while three studies were identified as theoretical or evaluation models.

The purpose of the 18 empirical studies was to survey or directly observe the reaction of stakeholders to curriculum integration strategies in order to identify or describe the relevant factors (Table 2). Study types included qualitative (*n* = 4) [11, 36–38], mixed-methods (*n* = 4) [39–42], quantitative survey (*n* = 4) [10, 43–45], and descriptive case studies (*n* = 6) [46–51]. Data collection methods included questionnaires (*n* = 9) [10, 39–45, 48], focus groups and small group interviews (*n* = 8) [11, 36–39, 41, 42, 48], system log analyses (*n* = 3) [44, 47, 48], direct observations (*n* = 1) [44], or narrative descriptions of experiences with integration (*n* = 5) [46, 47, 49–51]. The vast majority of studies reported experiences from integration of VPs into medical curricula (*n* = 15). Two studies reported integration of VPs into nursing programs [40, 51], one in a dentistry [40] and one in a pharmacy program [41]. One study was unspecific about the health professions program [46].

**Table 2:** Summary of characteristics of included studies – empirical studies

Study	Country	Study approach	Data collection	Study program	Year/phase of program	Medical topic	Type and number of participants	Name of VP system	Number of VPs	Source of VPs/ project; collection
Berman et al., 2009	USA	Quantitative	Survey (paper&pencil)	Medicine	3rd year	Pediatrics	545 students	CASUS	31	Collaborative dev./ CLIPP
Botezatu et al., 2010a	Columbia	Qualitative	Focus groups	Medicine	4th year	Internal medicine	16 students	Web-SP	ns	Self-developed
Botezatu et al., 2010b	Columbia, Sweden	Mixed-method	Survey	Medicine, nursing, dentistry	ns; undergrad.	Mixed	39 students, teachers, course directors, and university leaders	Web-SP	ns	Self-developed; data collected by students
Dafli et al., 2019	Greece	Quantitative	Survey (online)	Medicine	ns; undergrad.	ns	219 students, 33 teachers	Open-Labyrinth	59	Collaborative dev./ mEducator, ARIADNE
Dahri et al., 2019	Canada	Mixed-methods	Survey, focus groups	Pharmacy	1st -3rd year	Mixed	180 students in survey, 6 in focus group	VIC	4	Self-developed by students
Edelbring et al., 2011	Sweden	Qualitative	Semi-structured Interviews (individual, small group)	Medicine	3rd year	Rheumatology	31 students	ReumaCase	4	Self-developed
Edelbring et al., 2012	Sweden	Quantitative	Survey, system logs, observations	Medicine	1st year	Clinical clerkship introduction	247 students	Web-SP	4	Self-developed
Ellaway et al., 2015a	Canada	Descriptive	Case series	ns	ns; undergrad.	ns	ns	Open-Labyrinth	ns	Self-developed; PINE, eViP collections
Fors et al., 2009	Romania, Columbia, Sweden	Descriptive^(a)^	Case series	Medicine	undergrad., 5th year^(b)^	Internal medicine, surgery, endocrinology	22 students, otherwise not stated	Web-SP	3^(b)^, ns	Collaborative dev./ eViP
Hege et al., 2007	Germany	Descriptive	Case series, system logs	Medicine^(c)^	3rd year	Internal, occupational medicine	698 students^(c)^	CASUS	up to 10	Self-developed
Hirumi et al., 2016	USA	Descriptive^(c)^	Survey, focus groups, system logs	Medicine	2nd year	Neurology	119 students	NERVE	> 3	Self-developed
Huwendiek et al., 2013	Germany	Qualitative	Focus groups	Medicine	5th year	Pediatrics	39 students	Campus	15	Self-developed
Kassianos et al., 2023	United Kingdom	Qualitative	Semi-structured interviews (phone)	Medicine	undergrad.	Mixed	13 medical teachers	various/ not specified	ns	ns
Kelley et al., 2015	USA	Descriptive	Case study	Nursing	ns; undergrad.	Advanced assessment	ns	DCE	ns	External, commercial
Kulasegaram et al., 2018	Canada	Descriptive	Case study	Medicine	1st -2nd year	Mixed	518 students	ns	72	ns
Lang et al., 2013	USA, Canada	Quantitative	Survey (email)	Medicine	Internal med. clerkship	Internal medicine	33–45 clerkship directors	CASUS	36	Collaborative dev./ SIMPLE
McCarthy et al., 2015	United Kingdom	Mixed-methods	Survey study, focus groups	Medicine	3rd year	Microbiology	161 students in survey, 11 in focus group	Microbiology VP	7	ns
Schifferdecker et al., 2012	USA	Mixed-methods	Survey study, semi-structured interviews (phone)	Medicine	3rd year	Pediatrics	47 clerkship directors in survey, 11 individual interviews	CASUS	31	Collaborative dev./ CLIPP

*ns *not stated, *Undergrad*. Undergraduate

^(a)^experimental for the Romanian case study; ^(b)^excluded case study with law students; ^(c)^design-based research

The remaining three of the included studies represented a more theoretical approach: one aimed to create a conceptual model [25]; the other two [24, 26] presented evaluation models of the integration process (Table 3). We analysed them separately, considering their different structures, and we mapped the components of these models to our framework in the last stage of the framework synthesis.

**Table 3:** Summary of characteristics of included studies - theoretical and evaluation model studies

Authors, Year	Country	Study design	Constructed model, framework, instrument	Study program	Theoretical underpinning	Main identified conceptual domains; categories; factors	Validation method	VP system in test
Georg & Zary, 2014	Sweden	Framework design	vpNAM: virtual patient Nursing Activity Model	Nursing	• Not stated	• Introduction • Data collection • Assignment • Feedback • Reflection	Applied to the 3rd semester of the undergraduate nursing education program at Karolinska Institutet using VIC; *n* = 102 students	VIC
Kleinheksel & Ritzhaupt, 2017	USA	Instrument development	VPAIN: Virtual Patient Adoption and Integration in Nursing	Nursing	• Roger’s Diffusion of Innovation • Miller and Bull’s Factors Influencing Nurse Acadmic‘s Adoption of simulation	• Adoption: Worldbuilding, Pedagogy, Differentiation, Encouragement, Clarity, Evaluation, Administrative Pressure, Visibility; • Integration: Hour Replacement, Intensive Integration, Leveling, Preparation, Benchmarking	Exploratory Factor Analysis (survey completed by *n* = 178 nurse educators). The factors account for 53,3% of variance.	DCE
Huwendiek et al., 2009	Germany	Instrument development	eViP project Curricular Integration of Virtual Patients Questionnaire and Checklist	Medicine	• Community of Inquiry Model • Khan’s blended learning framework • Heyer’s criteria categorizing didactic scenarios	• Teaching presence • Cognitive presence • Social presence • Learning effect	Reviewed by eViP project partners and tested with students (no detail on the number of testing experts and students were given)	ns

### Themes in the developed framework

The developed framework (Table 4), which we named the iCoViP Virtual Patient Curriculum Integration Framework (iCoViP Framework), contains 14 themes and 51 topic codes. The final version of the codebook used in the study can be found in Supplementary Material S2. Below, we describe the individual themes.

**Table 4:** iCoViP Virtual Patient Curriculum Integration Framework

Kern’s step	Theme	Codes
1. General needs assessment	Goal	Memorable experience; Knowledge acquisition; Authenticity; Reflection; Safe learning environment; Clinical reasoning; Regulatory requirements; Patient-centred care
2. Targeted needs assessment	Phase	Early stage; Late stage
	Resources	Limitations’ awareness; VPs exchange; Teachers’ availability and effort
3. Goals and objectives	Alignment	Local specificity; Alignment strategies; Learning objectives; VP selection criteria
	Prioritisation	Mandatory strategy; Exam-relevance; Processing intensity; Adaptation; Visibility; Authenticity; Peer opinion; Time availability
4. Educational strategies	Relation	General; Expository methods; Simulations; Clinical teaching; Small groups; Optimal sequence; Free choice of learning methods
	Activities	Questions; Case presentations; Book referral; Similar case comparisons; Checklists; Lack of activities
5. Implementation	Time	Time allocation; Time efficiency; Deadlines
	Group	Group assignment; Individual assignments; Group size
	Presence	Face-2-face; Online learning; Blended learning
	Orientation	Faculty Development; Students
	Infrastructure	Stable Internet; Security; Usability; Interoperability; Robust software; IT support
6. Evaluating the effectiveness	Sustainability & Quality	Extension of existing VP collections; VP review & update; Student evaluation
	Assessment	Summative assessment; Feedback; Formative assessment; Learning analytics

### General needs assessment

#### Goal

In the *Goal* theme, we coded perceptions regarding appropriate general uses of VPs in curricula. This covers the competencies to be trained using VPs, but also unique strengths and limitations of VPs as a learning method that should influence decisions regarding their adoption in curricula.

A common opinion was that VPs should target clinical reasoning skills and subskills such as acquisition/organisation of clinical information, development of illness scripts (sign, symptoms, risk factors, knowledge of disease progress over time), patient-centred care (including personal preferences and cultural competencies in patient interaction) [11, 36–40, 42–46, 49–51]. According to these opinions, a strength of VPs is their potential for self-directed learning in an authentic, practice-relevant, safe environment that gives opportunities for reflection and “productive struggle” [37, 39, 49]. VPs also make it possible for students to practise decision-making in undifferentiated patient cases and observe the development of disease longitudinally [45]. For instance, some students valued the potential of VPs as a tool that integrates basic knowledge with clinical application in a memorable experience:

*We associate a disease more to a patient than to the textbook. If I saw the patient, saw the photo and questioned the patient in the program, I will remember more easily, I’ll have my flashback of that pathology more than if I only studied my class notes or a book. {Medical student, 4th year, Columbia}* [36].

Another perceived function of VPs is to help fill gaps in curricula and clinical experiences [36–38, 42, 45, 50]. This supporting factor for the implementation of VPs in curricula is particularly strong when combined with the need to meet regulatory requirements [42].

Varying opinions were expressed regarding the aim of VPs to represent rare diseases (or common conditions but with unusual symptoms) [43, 48] versus common clinical pictures [37, 40]. Another tension arose when considering whether VPs should be used to introduce new factual/conceptual knowledge versus serving as a knowledge application and revision tool:

*The students, however, differed from leaders and teachers in assuming that VPS should offer a reasonable load of factual knowledge with each patient. More as a surprise came the participants’ preference for usual presentations of common diseases.* [40].

Limitations of VPs were voiced when the educational goal was related to physical contact and hands-on training because, in some aspects of communication skills, physical examination, or application of medical equipment, VPs clearly have inferior properties to real patients, human actors or physical mannequins [36, 51].

### Targeted needs assessment

#### Phase

The *Phase* theme described the moment in curricula when the introduction of VPs was regarded as adequate. According to some opinions, VPs should be introduced early in curricula to provide otherwise limited exposure to real patients [39, 43]:

*Students of the pre-clinical years show a high preference in the adoption of VPs as learning activities. That could be explained from the lack of any clinical contact with real patients in their two first years of study and their willingness to have early, even virtual, clinical encounters.* [43].

The tendency to introduce VPs early in curricula was confronted with the problem of students’ limited core knowledge as they were required to use VPs before they had learnt about the features of the medical conditions they were supposed to recognise [41, 48]. At the other end of the time axis, we did not encounter opinions that specified when it would be too late to use VPs in curricula. Even final-year students stated that they preferred to augment their clinical experience with VPs [43].

#### Resources

In the *Resources* theme, we gathered opinions regarding the cost and assets required for the integration of VPs into curricula. Cost can be a barrier that, if not addressed properly, can slow down or even stop an implementation, therefore it should be addressed early in the implementation process. This includes monetary funds [42] and availability of adequately qualified personnel [38] and their time [47].

For instance, it was found that if a faculty member is primarily focused on clinical work, their commitment to introducing innovation in VPs will be limited and will tend to revert to previous practices unless additional resources are provided to support the change [38].

The *Resources* theme also included strategies to follow when there is only a limited number of resources to implement VPs in a curriculum. Some suggested solutions included the sharing of VPs with other institutions [50], the exchange of know-how on the implementation of VPs with more experienced institutions and networks of excellence [38, 42], and increasing faculties’ awareness of the benefits of using VPs, also in terms of reduced workload after the introduction of VPs in curricula [38]. Finally, another aspect of this theme was the (lack of) awareness of the cost of implementing VPs in curricula across stakeholder groups [40].

### Goals and objectives

#### Alignment

The *Alignment* theme grouped utterances highlighting the importance of selecting the correct VP content for curricula and matching VPs with several elements of curricula, such as learning objectives, the content of VPs across different learning forms, as well as the need to adapt VPs to local circumstances. The selection criteria included discussion regarding the number of VPs [36], fine-grained learning objectives that could be achieved using VPs [42, 50], and selection of an appropriate difficulty level, which preferably should gradually increase [11, 49].

It was noticed that VPs can be used to systematically cover a topic. For example, they can align with implementation of clinical reasoning themes in curricula [38] or map a range of diseases that are characteristic of a particular region of interest, thereby filling gaps in important clinical exposure and realistically representing the patient population [36].

Several approaches were mentioned regarding the alignment of VPs with curricula that include the selection of learning methods adjusted to the type of learning objectives [45], introduction of VPs in small portions in relevant places in curricula to avoid large-scale changes [38], alignment of VP content with assessment [39], and the visibility of this alignment by explicitly presenting the specific learning objectives addressed by VPs [49]. It is crucial to retain cohesion of educational content across a range of learning modalities:

*I worked through a VP, and then I went to the oncology ward where I saw a patient with a similar disease. After that we discussed the disease. It was great that it was all so well coordinated and it added depth and some [sic!] much needed repetition to the case. {Medical student, 5th year, Germany}* [11].

We also noted unresolved dilemmas, such as whether to present VPs in English as the modern *lingua franca* to support the internationalisation of studies, versus the need to adapt VPs to the local native language of learners in order to improve accessibility and perceived relevance [50].

#### Prioritisation

Several studies presented ideas for achieving higher *Prioritisation* of VPs in student agendas. The common but “heavy-handed” approach to increase motivation was to make completion of VPs a mandatory requirement to obtain course credits [36, 48, 51]. However, this approach was then often criticised for promoting superficial learning and lack of endorsement for self-directed learning [47]. Motivation was reported to increase when content was exam-relevant [11].

According to yet another mentioned strategy, motivation comes with greater engagement of teachers who intensively reference VPs in their classes and often give meaningful feedback regarding their use [40] or construct group activities around them [46]. It was suggested that VPs ought to have dedicated time for their use which should not compete with activities with obviously higher priorities, such as meeting real patients [37].

Another idea for motivation was adjustment of VPs to local needs, language and culture. It was indicated that it would be helpful to promote VPs’ authenticity by stressing the similarity of presented scenarios to problems clinicians encounter in clinical practice (e.g., using teacher testimonials [48]). Some students saw VPs as being more relevant when they are comprehensively described in course guides and syllabi [39]. The opinions about VPs that circulate among more-experienced students are also important:

*Definitely if the year above kind of approves of something you definitely think you need it. {Medical student, 3rd year, UK}* [39].

Peer opinion was also important for teachers, who were reported to be more likely to adopt VPs in their teaching if they have heard positive opinions from colleagues using them, know the authors of VP cases, or respect organisations that endorse the use of VP software [38, 42]:

*I was amazed because it was a project that seemed to have incredible scope, it was huge. I was impressed that there was the organization to really roll out and develop all these cases and have this national organization involved. {Clerkship director, USA}* [42].

### Educational strategies

#### Relation

The *Relation* theme contained opinions about the connections between VPs and other types of learning activities. This theme was divided into preferences regarding which types of activities should be replaced or extended by VPs, and the relative order in which they should appear in curricula. We noticed general warnings that VPs should not be added on top of existing activities as this is likely to cause work overload for students [10, 45]. The related forms of education that came up in the discussions were expository methods like lectures and reading assignments (e.g., textbooks, websites), small group discussions in seminars (e.g., problem-based learning [PBL] sessions, follow-up seminars), alternative forms of simulations (e.g., simulated patients, human patient simulators), clinical teaching (i.e., meeting with real patients and bedside learning opportunities), and preparation for assessments.

Lectures were seen as a form of providing core knowledge that could later be applied in VPs:

*Working through the VP before attending the lecture was not as useful to me as attending the lecture before doing the VP. I feel I was able to get more out of the VP when I first attended the lecture in which the substance and procedures were explained. {Medical student, 5th year, Germany}* [11].

Textbooks were helpful as a source of reference knowledge while solving VPs that enabled students to reflect while applying this knowledge in clinical context. Such a learning scenario was regarded impossible in front of real patients:

*But here it’s very positive right now when we really don’t know everything about rheumatic diseases, that we can sit with our books at the same time as we have a patient in front of us. {Medical student, 3rd year, Sweden}* [37].

Seminars (small group discussions) were perceived as learning events that motivate students to work intensively with VPs and as an opportunity to ask questions about them [11, 46, 47], with the warning that teachers should not simply repeat the content of VPs as this would be boring [44]. The reported combination of VPs with simulated patients made it possible to increase the fidelity of the latter by means of realistic representation of clinical signs (e.g., cranial nerve palsies) [48]. It was noticed that VPs can connect different forms of simulation, “turn[ing] part-task training into whole-task training” [46], or allow more thorough and nuanced preparation for other forms of simulation (e.g., mannequin-based simulation) [46]. A common thread in the discussion was the relation between VPs and clinical teaching [10, 11, 37, 39, 45, 46]. The opinions included warnings against spending too much time with VPs at the expense of bedside teaching [37, 51]. The positive role of VPs was highlighted in preparing for clinical experience or as a follow-up to meeting real patients because working with VPs is not limited by time and is not influenced by emotions [37].

Huwendiek et al. [11] suggested a complete sequence of activities which has found confirmation in some other studies [48]: lectures, VP, seminars and, finally, real patients. However, we also identified alternative solutions, such as VPs that are discussed between lectures as springboards to introduce new concepts [49]. In addition, some studies concluded that students should have the right to decide which form of learning they prefer in order to achieve their learning objectives [38, 48], but this conflicts with limited resources, a problem the students seem not to consider when expressing their preferences.

#### Activities

In the *Activities* theme, we grouped statements about tasks constructed by teachers around VPs. This includes teachers asking questions to probe whether students have understood the content of VPs, and guiding students in their work with VPs [11, 49]. Students were also expected to ask their teachers questions to clarify content [43]. Some educators felt that students trained using VPs ask too many questions instead of relying more on their clinical reasoning skills and asking fewer, but more pertinent questions [38].

Students were asked to compare two or more VPs with similar symptoms to recognise key diagnostic features [11] and to reflect on cases, discuss their decisions, and summarise VPs to their peers or document them in a standardised form [11, 46, 49, 51]. Another type of activity was working with textbooks while solving VP cases [37] or following a standard/institutional checklist [51]. Finally, some students expected more activities around VPs and felt left alone to struggle with learning with VPs [37].

### Implementation

#### Time

Another theme grouped stakeholders’ opinions regarding *Time.* A prominent topic was the time required for VP activities. Some statements provided the exact amount of time allocated to VP activities (e.g., one hour a week [51]), sometimes suggesting that it should be increased. There were several comments from students complaining about insufficient time allocated for VP activities:

*There was also SO much information last week and with studying for discretionary IRATs constantly, I felt that I barely had enough time to synthesize the information and felt burdened by having a deadline for using the simulation. {Medical student, 2nd year, USA}* [48].

Interestingly, the perceived lack of time was sometimes interpreted by researchers as a matter of students not assigning high enough priority to VP tasks because they do not consider them relevant [39].

Some students expected their teachers to help them with time management. Mechanisms for this included explicitly allocated time slots for work with VPs, declaration of the required time spent on working with VPs, and setting deadlines for task completion:

*Without a time limit we can say: I’ll check the cases later, and then nothing happens; but if there’s a time limit, well, this week I see cardiac failure patients etc. It’s more practical for us and also for the teachers, I think. {Medical student, 4th year, Columbia}* [36].

This expectation conflicts with the views that students should learn to self-regulate their activities, that setting a minimum amount of time that students should spend working with VPs will discourage them from doing more, and that deadlines cause an acute burst of activity shortly before them, but no activity otherwise [47, 48].

Finally, it was interesting to notice that some educators and students perceived VPs as a more time-efficient way of collecting clinical experience than meeting real patients [37, 38].

#### Group

The *Group* theme included preferences for working alone or in a group. The identified comments revealed tensions between the benefits of working in groups, such as gaining new perspectives, higher motivation thanks to teamwork, peer support:

*You get so much more from the situation when you discuss things with someone else, than if you would be working alone. {Medical student, 3rd year, Sweden}* [37].

and the flexibility of working alone [43, 44, 46, 49]. Some studies reported on their authors’ experiences in selection of group size [11, 48]. It was also noted that smaller groups motivated more intensive work [41, 44].

#### Presence

In the *Presence* theme, we coded preferences regarding whether students should work on VPs in a computer lab, a shared space, seminar rooms, or at home. Some opinions valued flexibility in selecting the place of work (provided a good internet connection is available) [11, 36]. Students reported working from home in order to prepare well for work in a clinical setting:

*... if you can work through a VP at home, you can check your knowledge about a certain topic by working through the relevant VP to see how you would do in a more realistic situation. {Medical student, 5th year, Germany}* [11].

Some elements of courses related to simulated patient encounters had to be done during obligatory face-to-face training in a simulation lab (e.g., physical examination) that accompanied work with VPs [51]. Finally, it was observed that VPs offer sufficient flexibility to support different forms of blended learning scenarios [46]. Synchronous collaborative learning can be combined with asynchronous individual learning, which is particularly effective when there is a need for collaboration between geographically dispersed groups [46], for instance if a school has more than one campus.

#### Orientation

In the *Orientation* theme, we included all comments that relate to the need for teacher training, the content of teacher training courses, and the form of preparation of faculty members and students for using VPs. Knowledge and skills mentioned as useful for the faculty were awareness about how VPs fit into curricula [42], small-group facilitation skills, clinical experience [11], and experience with online learning [38]. Teachers expected to be informed about the advantages/disadvantages and evidence of effectiveness of VPs [38]. For students, the following prerequisites were identified: the ability to operate VP tools and experience with online learning in general, high proficiency of the language in which the VPs are presented and, for some scenarios (e.g., learning by design), also familiarity with VP methodology [38, 47, 48, 50, 51]. It was observed that introduction of VPs is more successful when both teachers and students are familiar with the basics of clinical reasoning theory and explicit teaching methods [38].

Forms of student orientation that were also identified regarding the use of VPs included demonstrations and introductions at the start of learning units [42], handouts and email reminders, publication of online schedules for assigned VPs, and expected time to complete them [11, 48].

#### Infrastructure

The *Infrastructure* theme grouped stakeholders’ requirements regarding the technical environment in which VPs work. This included the following aspects: stable internet connection, secure login, usability of the user interface, robust software (well tested for errors and able to handle many simultaneous users), interoperability (e.g., support for the standardised exchange of VPs between universities) and access to an IT helpdesk [11, 40, 42, 47, 50]. It was noticed that technical glitches can have a profound influence on the perceived success of VP integration:

*Our entire team had some technical difficulties, whether during the log-in process or during the patient interviews themselves and felt that our learning was somewhat compromised by this. {Medical student, 2nd year, USA}* [48].

### Evaluating the effectiveness

#### Sustainability & quality

In the *Sustainability & Quality* theme, we indexed statements regarding the need to validate and update VP content, and its alignment with curricular goals and actual assessment to respond to changes in local conditions and regulatory requirements [45].

The need to add new cases to VP collections that are currently in use was mentioned [40]. This theme also included the requirement to evaluate students’ opinions on VPs using questionnaires, feedback sessions and observations [47–49]. Some of the stakeholders required evidence regarding the quality of VPs before they decided to adopt them [38, 42, 50]. Interestingly, it was suggested that awareness of the need for quality control of VPs varied between stakeholder groups, with low estimation of the importance of this factor among educational leaders:

*Leaders also gave very low scores to both case validation and case exchange with other higher education institutions (the latter finding puts into perspective the current development of VPS interoperability standards). The leaders’ lack of interest in case validation may reflect a de facto conviction, that it is the ‘shell’ that validates the content.* [40].

#### Assessment

The *Assessment* theme encompasses a broad selection of topics related to various forms of using VPs in the assessment of educational outcomes related to VPs. This includes general comments on VPs as an assessment form, use of VPs in formative and summative assessment, as well as the use of learning analytics methods around VPs.

General topics identified in this theme included which learning objectives should be assessed with VPs, such as the ability to conduct medical diagnostic processes effectively [36], the authenticity of VPs as a form of examination [36], the use of VPs for self-directed assessment [11, 39, 43, 46], and the emotions associated with assessment using VPs, e.g., reduced stress and a feeling of competitiveness [36, 48].

Other topics discussed in the context of assessment included the pedagogical value of using VPs for assessments [36], such as the improved retention of information through reflection on diagnostic errors made with VPs [48], and VPs’ ability to illustrate the consequences of students’ errors [46]. Methods of providing feedback during learning with VPs were also described [11]. It was highlighted that data from assessments using VPs can aid teachers in planning future training [49, 51]. Furthermore, it was observed that feedback from formative assessments with VPs motivates students to engage more deeply in their future learning [10, 41, 47]:

*It definitely helped what we did wrong and what we should have caught, because there was a lot that I missed and I didn’t realize it until I got the feedback and in the feedback it also said where you would find it most of the time and why you would have looked there in the first place. {Pharmacy student, 4th year, Canada}* [41].

In several papers [42, 47, 48, 51], suggestions were made regarding the types of metrics that can be used to gauge students’ performance (e.g., time to complete tasks related to VPs, the accuracy of answers given in the context of VPs, recall and precision in selecting key features in the diagnostic process, the order of selecting diagnostic methods, and the quality of medical documentation prepared by students from VPs). The use of specific metrics and the risks associated with them were discussed. For instance, time spent on a task was sometimes seen as a metric of decision efficiency (a speed-based decision score) that should be minimised [48], or as an indicator of diligence in VP analysis that should be maximised [47]. Time measurements in on-line environments can be influenced by external factors like parallel learning using different methods (e.g. consulting a textbook) or interruptions unrelated to learning [47].

Finally, the analysed studies discussed summative aspects of assessment, including arguments regarding the validity of using VPs in assessments [51], the need to ensure alignment between VPs and examination content [49], and the importance of VP assessment in relation to other forms of assessment (e.g., whether it should be part of high-stakes examinations) [40, 51]. The studies also explored forms of assessment that should be used to test students’ assimilation of content delivered through VPs [47], the challenges related to assessing clinical reasoning [38], and the risk of academic dishonesty in grading based on VP performance [48].

### Mapping of the literature using the developed framework

**Fig. 2:**
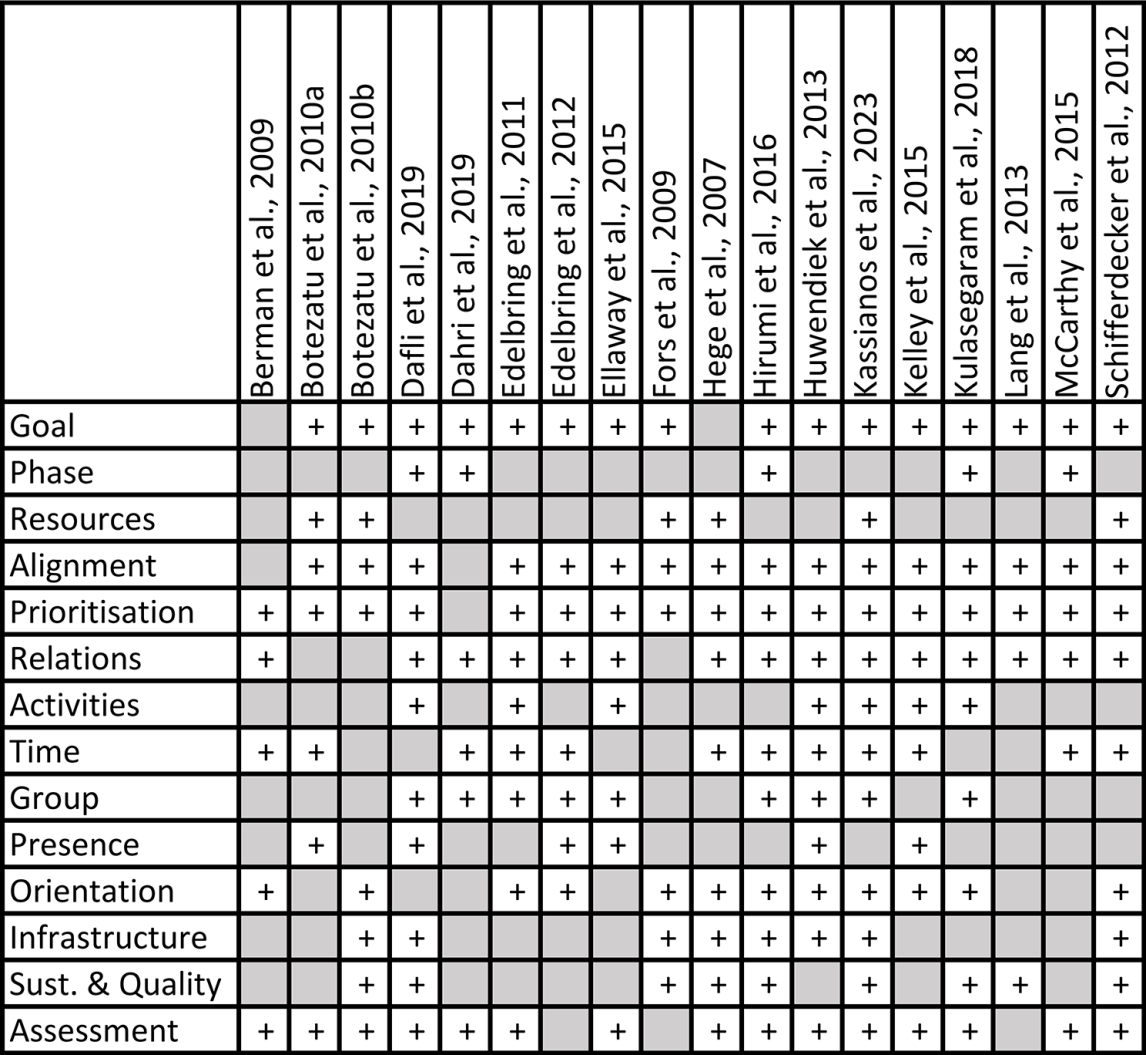
Code matrix of the occurrence of themes in the included empirical studies

We mapped the occurrence of the iCoViP Framework themes across the included empirical studies, as presented in Fig. 2.

Table 5 displays a pooled number of studies in which each theme occurred. The three most frequently covered themes were *Prioritisation*, *Goal*, and *Alignment*. These themes were present in approx. 90% of the analysed papers. Each theme from the framework appeared in at least four studies. The least-common themes, present in fewer than one-third of studies, were *Phase*, *Presence*, and *Resources*.

**Table 5:** Frequency of occurrence of iCoViP Framework themes in included studies

Code	Articles	% articles
Prioritisation	17	94%
Alignment	16	89%
Goal	16	89%
Assessment	15	83%
Relations	15	83%
Orientation	12	67%
Time	12	67%
Group	9	50%
Sustainability & Quality	9	50%
Infrastructure	8	44%
Activities	7	39%
Presence	6	33%
Resources	6	33%
Phase	5	28%

**Fig. 3:**
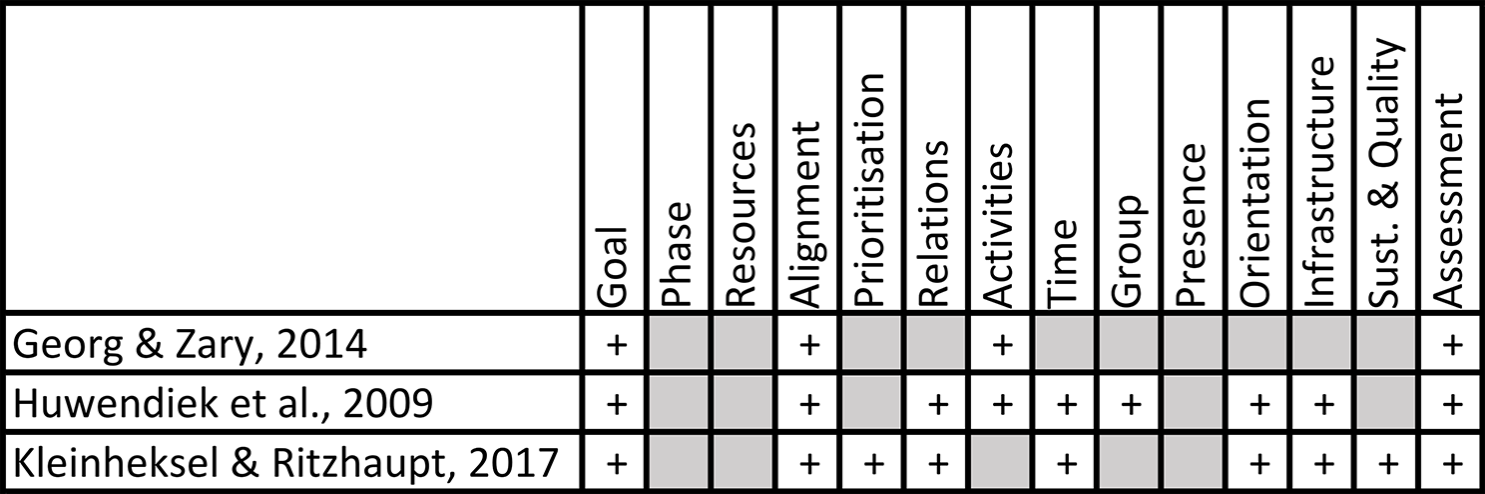
Mapping of the existing integration models to the iCoViP Framework

We mapped the iCoViP Framework to the three identified existing theoretical and evaluation models (Fig. 3).

None of the compared models contained a category that could not be mapped to the themes from the iCoViP Framework. The model by Georg & Zary [25] covered the fewest themes from our framework, including only the common categories of *Goal, Alignment, Activities* and *Assessment*. The remaining two models by Huwendiek et al. [24] and Kleinheksel & Ritzhaupt [26] underpinned integration quality evaluation tools and covered the majority of themes (9 out of 14 each). There were three themes not covered by any of the models: *Phase, Resources, and Presence*.

### Quality assessment of studies

The details of the quality appraisal of the empirical studies using the QuADS tool are presented in Supplementary Material S3. The rated papers had medium (50–69%; [39, 40, 43]) to high quality (≥ 70%; [10, 11, 36–38, 41, 42, 44, 45]). Owing to the difficulty in identifying the study design elements in the included descriptive case studies [46–51], we decided against assessing their methodological quality with the QuADS tool. This difficulty can also be interpreted as indicative of the low quality of the studies in this group.

The QuADS quality criterion that was most problematic in the reported studies was the inadequate involvement of stakeholders in study design. Most studies reported the involvement of students or teachers only in questionnaire pilots, but not in the conceptualisation of the research. Another issue was the lack of explicit referral to the theoretical frameworks upon which the studies were based. Finally, in many of the studies, participants were selected using convenience sampling, or the authors did not report purposeful selection of the study group.

We found high-quality studies in qualitative, quantitative, and mixed-methods research. There was no statistical correlation between study quality and the number of topics covered. For sensitivity analysis, we excluded all medium-quality and descriptive studies from the analysis; this did not reduce the number of iCoViP Framework topics covered by the remaining high-quality studies.

## Discussion

In our study, we synthesised the literature that describes stakeholders’ perceptions of the implementation of VPs in health professions curricula. We systematically analysed research reports from a mix of study designs that provided a broad perspective on the relevant factors. The main outcome of this study is the iCoViP Framework, which represents a mosaic of 14 themes encompassing many specific topics encountered by stakeholders when reflecting on VPs in health professions curricula. We examined the prevalence of the identified themes in the included studies to justify the relevance of the framework. Finally, we assessed the quality of the analysed studies.

### Significance of the results

The significance of the developed framework lies in its ability to provide the health professions education community with a structure that can guide VP implementation efforts and serve as a scaffold for training and research in the field of integration of VPs in curricula. The developed framework was immediately applied in the structuring of the iCoViP Curriculum Implementation Guideline. This dynamic document, available on the website of the iCoViP project [https://icovip.eu/knowledge-base], presents the recommendations taken from the literature review and the project partners’ experiences with how to implement VPs, particularly the collection of 200 VPs developed during the iCoViP project [23]. To improve the accessibility of this guideline, we have added a glossary with definitions of important terms. We have already been using the framework to structure faculty development courses on the topic of teaching with VPs.

It is clear from our study that the success of integrating VPs into curricula depends on the substantial effort that is required of stakeholders to make changes in the learning environment to enable VPs to work well in the context of local health professions education programs. The wealth of themes discussed in the literature around VPs confirms what is known from implementation science: the quality of the implementation is as important as the quality of the product [15]. This might be disappointing for those who hope VPs are a turnkey solution that can be easily purchased to save time, under the misconception that implementation will occur effortlessly.

Our review also makes it evident that implementation of VPs is a team endeavour. Without understanding, acceptance and mutual support at all levels of the institutional hierarchy and a broad professional background, different aspects of the integration of VPs into curricula will not match. Students should not be left to their own devices when using VPs. They need to understand the relevance of the learning method used in a given curriculum by observing teachers’ engagement in the routine use of VPs, and they should properly understand the relationship between VPs and student assessment. Despite the IT-savviness of many students, they should be shown how and when to use VPs, while also allowing room for creative, self-directed learning. Finally, students should not get the impression that their use of VPs comes at the expense of something they give higher priority, such as direct patient contact or teacher feedback. Teachers facilitating learning with VPs should be convinced of their utility and effectiveness, and they need to know how to use VPs by themselves before recommending them to students. It is important that teachers are aware that VPs, like any other teaching resources, require quality control linked with perpetual updates. They should feel supported by more-experienced colleagues and an IT helpdesk if methodological or technical issues arise. Last but not least, curriculum managers should recognise the benefits and limitations of VPs, how they align with institutional goals, and that their adoption requires both time and financial resources for sustainment. All of this entails communication, coordinated efforts, and shared decision-making during the implementation of VPs in curricula.

### Implications for the field

Per Nilsen has divided implementation theories, models and frameworks into three broad categories: process models, determinant frameworks and evaluation models [16]. We view the iCoViP Framework primarily as a process model. This perspective originates from the initial framework we adopted in our systematic review, namely Kern’s 6-steps curriculum development process [30], which facilitates the grouping of curricula integration factors into discrete steps and suggests a specific order in which to address implementation tasks. Our intention in using this framework was also to structure how-to guidelines, which are another hallmark of process models. As already noted by Nilsen and as is evident in Kern’s model, implementation process models are rarely applied linearly in practice and require a pragmatic transition between steps, depending on the situation.

The boundary between the classes of implementation models is blurred [16] and there is significant overlap. It is therefore not surprising that the iCoViP framework can be interpreted through the lens of a determinant framework which configures many factors (facilitators and barriers) that influence VP implementation in curricula. Nilsen’s category of determinant frameworks includes the CFIR framework [52], which was also chosen by Kassianos et al. to structure their study included in this review [38]. A comparison of the themes emerging from their study and our framework indicates a high degree of agreement (as depicted in Fig. 2). We interpret this as a positive indication of research convergence. Our framework extends this research by introducing numerous fine-grained topic codes that are characteristic of VP integration into curricula.

The aim of our research was not to develop an evaluation framework. For this purpose, the two evaluation tools available in the literature by Huwendiek et al. [24] and Kleinheksel & Ritzhaupt [26] are suitable. However, the factors proposed in our framework can further inform and potentially extend existing or new tools for assessing VP integration.

Despite the plethora of available implementation science theories and models [16], their application in health professions curricula is limited [15]. The studies included in the systematic review only occasionally reference implementation sciences theories directly (exceptions are CFIR and UTAUT [38], Rogers’ Diffusion of Innovation Theory [26, 42] and Surry’s RIPPLES model [42]). However, it is important to acknowledge that implementation science is itself an emerging field that is gradually gaining recognition. Furthermore, as noticed by Dubrowski & Dubrowski [17], the direct application of general implementation science models does not guarantee success and requires verification and adaptation.

### Limitations and strengths

This study is based on stakeholders’ perceptions of the integration of VPs into curricula. The strength of the evidence behind the recommendations expressed in the analysed studies is low from a positivist perspective as it is based on subjective opinions. However, by adopting a more interpretivist stance in this review, our goal is not to offer absolute, ready-to-copy recommendations. Instead, we aim to provide a framework that organises the implementation themes identified in the literature into accessible steps. It is beyond the scope of this review to supply an inventory of experimental evidence for the validity of the recommendations in each topic, as was intended in previous systematic reviews [4]. We recognise that, for some themes, it will always be challenging to achieve a higher level of evidence due to practical constraints in organising studies that experiment with different types of curricula. The complexity, peculiarities, and context-dependency of implementation likely preclude one-size-fits-all recommendations for VP integration. Nevertheless, even in such a situation, a framework for sorting through past experiences with integration of VPs proves valuable for constructing individual solutions that fit a particular context.

The aim of our study was to cover experiences from different health professions programs in the literature synthesis. However, with a few exceptions, the results show a dominance of medical programs in research on VP implementation in curricula. This, although beyond the authors’ control, limits the applicability of our review findings. The data clearly indicates a need for more research into the integration of VPs into health professions curricula other than medicine.

The decision to exclude single-factor studies from the framework synthesis is justified by our aim to provide a comprehensive overview of the integration process. Nevertheless, recommendations from identified single-factor studies [53–55] were subsequently incorporated into the individual themes in the iCoViP project implementation guideline. We did not encounter any studies on single factors that failed to align with any of the identified themes within the framework. Due to practical reasons concerning the review’s feasibility, we did not analyse studies in languages other than English and did not explore non-peer-reviewed grey literature databases. However, we recognise the potential of undertaking such activities in preparing future editions of the iCoViP guideline as we envisage this resource as an evolving document.

We acknowledge that our systematic review was shaped by the European iCoViP project [23]. However, we did not confine our study to just a single VP model, thereby encompassing a broad range of technical implementations. The strength of this framework synthesis lies in the diversity of its contributors affiliated with several European universities in different countries, who were at different stages of their careers, and had experience with various VP systems.

### Further research

The iCoViP framework, by charting a map of themes around VP integration in health professions curricula, provides a foundation for further, more focused research on individual themes. The less-common themes or conflicts and inconsistencies in recommendations found in the literature synthesis may be a promising starting point.

An example of this is the phase of the curriculum into which a given VP fits. We see that proponents of early and late introduction of VPs use different arguments. The recommendation that VPs should be of increasing difficulty seems to be valid, but what is missing is the detail of what this means in practice. We envisage that this will be researched by exploring models of integration that cater for different levels of student expertise.

There are also varying opinions between those who see VPs as tools for presenting rare, intriguing cases, and those who see the commonality and practice relevance of the clinical problems presented in VPs as the most important factor. However, these opposing stances can be harmonised by developing a methodology to establish a well-balanced case-mix of VPs with different properties depending upon the needs of the learners and curricular context. Another point of division is the recognition of VPs as a tool for internationalising studies and supporting student mobility, versus the expectation that VPs should be adapted to local circumstances. These disparate beliefs can be reconciled by research into the design of activities around VPs that explicitly addresses the different expectations and confirm or refute their usefulness.

A significant barrier to the adoption of VPs is cost. While universities are occasionally willing to make a one-off investment in VPs for prestige or research purposes, the field needs more sustainable models. These should be suitable for different regions of the world and demonstrate how VPs can be maintained at a high level of quality in the face of limited time and resources. This is particularly important in low-resource countries and those affected by crises (e.g., war, natural disasters, pandemics), where the need for VPs is even greater than in developed countries due to the shortage of health professionals involved in teaching [56]. However, most of the studies included in our systematic review are from high-income countries. This shows a clear need for more research into the implementation of VPs in health professions curricula in developing countries.

Finally, an interesting area for future research is the interplay of different types of simulation modalities in curricula. The studies we reviewed do not recommend one type of simulation over another as each method has its unique advantages. In line with previous suggestions [46], we see a need for further research into practical implementation methods of such integrated simulation scenarios in curricula.

## Conclusions

Stakeholders’ perceptions were structured into 14 themes by this framework synthesis of mixed methods studies on the curricular integration of VPs. We envision that teachers, course directors and curriculum designers will benefit from this framework when they decide to introduce VPs in their teaching. We anticipate that our summary will inspire health professions education researchers to conduct new studies that will deepen our understanding of how to effectively and efficiently implement VPs in curricula. Last but not least, we hope that our research will empower students to express their expectations regarding how they would like to learn with VPs in curricula, thus helping them to become better health professionals in the future.

### Electronic supplementary material

Below is the link to the electronic supplementary material.

Supplementary Material 1

Supplementary Material 2

Supplementary Material 3

Supplementary Material 4

## Data Availability

All datasets produced and analysed during the current study are available from the corresponding author upon reasonable request.

## Abbreviations

VPs: Virtual patients
iCoViP: International Collection of Virtual Patients
QuADS: Quality Assessment with Diverse Studies
ED2: Liaison Committee on Medical Education (LCME) accreditation standard
CLIPP: Computer-assisted Learning in Paediatrics Program
PBL: Problem-Based Learning
