# Supplementary material for: Integrating virtual patients into undergraduate health professions curricula: a framework synthesis of stakeholders’ opinions based on a systematic literature review
Source: BMC Med Educ. 2024 Jul 5;24:727. doi: 10.1186/s12909-024-05719-1 (PMC11225252; doi:10.1186/s12909-024-05719-1)
Supplement: Supplementary file 1 — Supplementary Material 1 [file 12909_2024_5719_MOESM1_ESM.docx]

# Additional file 1: Search strategies

## PubMed/Medline

#1: Profession

medic*[tiab] OR

interprofessional*[tiab] OR

pharmacy[tiab] OR

nurs*[tiab] OR

clinical[tiab] OR

midwi*[tiab] OR

physio*[tiab] OR

dent*[tiab] OR

nutrition*[tiab] OR

diet*[tiab] OR

veterinar*[tiab] OR

health*[tiab] OR

occupational therapy[tiab]

#2: Education

student*[tiab] OR

educat*[tiab] OR

teacher*[tiab] OR

learn*[tiab] OR

undergraduate*[tiab] OR

tutor*[tiab] OR

instructor*[tiab]

#3:

"education, medical, undergraduate"[MeSH Major Topic]

#4: Virtual patients

virtual patient*[tiab] OR

patient scenario*[tiab] OR

interactive case*[tiab] OR

online patient*[tiab] OR

on-line patient*[tiab] OR

case-based e-learning[tiab] OR

case-based learning[tiab] OR

interactive scenario*[tiab]

#5: integration

integrat*[tiab] OR

curricul*[tiab] OR

implement*[tiab] OR

adopt*[tiab] OR

students use*[tiab] OR

employ*[tiab] OR

training scenario*[tiab] OR

study program*[tiab] OR

incorpo*[tiab] OR

embed*[tiab]

#6: time

2000/01/01:3000/12/31[Date - Publication]

# Final query

((#1 AND #2) OR #3) AND #4 AND #5 AND #6

## Embase

((medic* OR interprofessional* OR pharmacy OR nurs* OR clinical OR midwi* OR physio* OR dent* OR nutrition* OR diet* OR veterinar* OR health* OR ‘occupational therapy’):ti,ab) AND

((student* OR educat* OR teacher* OR learn* OR undergraduate* OR tutor* OR instructor*):ti,ab) AND

(('virtual patient*' OR 'patient scenario*' OR 'interactive case*' OR 'on$line?patient*' OR 'case-based e-learning' OR 'case-based learning' OR 'interactive scenario*'):ti,ab) AND

((integrat* OR curricul* OR implement* OR adopt* OR 'students use$' OR employ* OR 'training scenario$' OR 'study program*' OR incorpo* OR embed*):ti,ab) AND

[2022-2023]/py

[2000-2022]/py

## ERIC/CINAHL via EBSCOHost

(TI("medic*" OR "interprofessional*" OR "pharmacy" OR "nurs*" OR "clinical" OR "midwi*" OR "physio*" OR "dent*" OR "nutrition*" OR "diet*" OR "veterinar*" OR "health*" OR "occupational therapy")

OR AB("medic*" OR "interprofessional*" OR "pharmacy" OR "nurs*" OR "clinical" OR "midwi*" OR "physio*" OR "dent*" OR "nutrition*" OR "diet*" OR "veterinar*" OR "health*" OR "occupational therapy"))

AND

(TI("student*" OR "educat*" OR "teacher*" OR "learn*" OR "undergraduate*" OR "tutor*" OR "instructor*")

OR AB("student*" OR "educat*" OR "teacher*" OR "learn*" OR "undergraduate*" OR "tutor*" OR "instructor*"))

AND

(TI("virtual patient*" OR "patient scenario*" OR "interactive case*" OR "online#patient*" OR "case-based e-learning" OR "case-based learning" OR "interactive scenario*") OR

AB("virtual patient*" OR "patient scenario*" OR "interactive case*" OR "online#patient*" OR "case-based e-learning" OR "case-based learning" OR "interactive scenario*"))

AND

(TI("integrat*" OR "curricul*" OR "implement*" OR "adopt*" OR "students use*" OR "employ*" OR "training scenario*" OR "study program*" OR "incorpo*" OR "embed*") OR

AB("integrat*" OR "curricul*" OR "implement*" OR "adopt*" OR "students use*" OR "employ*" OR "training scenario*" OR "study program*" OR "incorpo*" OR "embed*")))

## Web of Science

#1

(AB=("medic*") OR TI=("medic*")) OR

(AB=("interprofessional*") OR TI=("interprofessional*")) OR

(AB=("pharmacy") OR TI=("pharmacy")) OR

(AB=("nurs*") OR TI=("nurs*")) OR

(AB=("clinical") OR TI=("clinical")) OR

(AB=("midwi*") OR TI=("midwi*")) OR

(AB=("physio*") OR TI=("physio*")) OR

(AB=("dent*") OR TI=("dent*")) OR

(AB=("nutrition*") OR TI=("nutrition*")) OR

(AB=("diet*") OR TI=("diet*")) OR

(AB=("veterinar*") OR TI=("veterinar*")) OR

(AB=("health*") OR TI=("health*")) OR

(AB=("occupational therapy") OR TI=("occupational therapy"))

#2

(AB=("student*") OR TI=("student*")) OR

(AB=("educat*") OR TI=("educat*")) OR

(AB=("teacher*") OR TI=("teacher*")) OR

(AB=("learn*") OR TI=("learn*")) OR

(AB=("undergraduate*") OR TI=("undergraduate*")) OR

(AB=("tutor*") OR TI=("tutor*")) OR

(AB=("instructor*") OR TI=("instructor*"))

#3

(AB=("virtual patient*") OR TI=("virtual patient*")) OR

(AB=("patient scenario*") OR TI=("patient scenario*")) OR

(AB=("interactive case*") OR TI=("interactive case*")) OR

(AB=("on$line patient*") OR TI=("on$line patient*")) OR

(AB=("case-based e-learning") OR TI=("case-based e-learning")) OR

(AB=("case-based learning") OR TI=("case-based learning")) OR

(AB=("interactive scenario*") OR TI=("interactive scenario*"))

#4

(AB=("integrat*") OR TI=("integrat*")) OR

(AB=("curricul*") OR TI=("curricul*")) OR

(AB=("implement*") OR TI=("implement*")) OR

(AB=("adopt*") OR TI=("adopt*")) OR

(AB=("students use*") OR TI=("students use*")) OR

(AB=("employ*") OR TI=("employ*")) OR

(AB=("training scenario*") OR TI=("training scenario*")) OR

(AB=("study program*") OR TI=("study program*")) OR

(AB=("incorpo*") OR TI=("incorpo*")) OR

(AB=("embed*") OR TI=("embed*"))

2022/01/01:3000/12/31[Date - Publication]

2000/01/01:3000/12/31[Date - Publication]
