# Supplementary material for: Integrating virtual patients into undergraduate health professions curricula: a framework synthesis of stakeholders’ opinions based on a systematic literature review
Source: BMC Med Educ. 2024 Jul 5;24:727. doi: 10.1186/s12909-024-05719-1 (PMC11225252; doi:10.1186/s12909-024-05719-1)
Supplement: Supplementary file 2 — Supplementary Material 2 [file 12909_2024_5719_MOESM2_ESM.docx]

**Additional file 2: Codebook used in the development of the iCoViP Framework**

| **Theme** | **Category** | **Definition** | **Representative quote for the category** | **Source of quote** |
| --- | --- | --- | --- | --- |
| 1. Goal | 1.1. Memorable experience | Emotional component of learning with VPs, that makes retaining knowledge easier | "We associate a disease more to a patient than to the textbook. If I saw the patient, saw the photo and questioned the patient in the program, I will remember more easily, I’ll have my flashback of that pathology, more than if I only studied my class notes or a book" (student 4). | Botezatu et al., 2010a |
| 1. Goal | 1.2. Knowledge acquisition | Using VPs to gain new knowledge | "Over the two years of the study, teaching knowledge and clinical reasoning remained the most important reasons for adopting VPs in the clerkship. The effectiveness of VPs in meeting these cognitive objectives is well supported in the medical education literature." | Lang et al., 2013 |
| 1. Goal | 1.3. Authenticity | Realistic presentation of patient cases and their context | "The students understand VPS as a “preparation for the real life as doctors”." | Botezatu et al., 2010a |
| 1. Goal | 1.4. Reflection | Opportunity to analyse a problem, draw conclusions | "The students felt that the VPs stimulated reflection on clinical work." | Edelbring et al., 2011 |
| 1. Goal | 1.5. Safe learning environment | The need of students to work in a non-judgmental environment that permits making mistakes and learning through errors | "Through virtual patients, students valued being able to make trial recommendations in a safe environment prior to their experiential practicums where suboptimal decision-making can have serious consequences." | Dahri et al., 2019 |
| 1. Goal | 1.6. Clinical reasoning | Knowledge integration and application, representation of diseases and its features, illness scripts, differential diagnosis, organisation of clinical knowledge, decision making and problem solving. | "It is important for students to study VP tasks because this enhances learning and clinical reasoning skills, which are important for students’ future medical practice." | Huwendiek et al., 2013 |
| 1. Goal | 1.7. Regulatory requirements | Consistency of teaching across a country to meet accreditation standards, but also to fill gaps in clinical exposure. This may be a local issue, which affects particular students (make-up activity to ensure completion of learning objectives). | "It ensures that you get to see these typical patients. If you’re not fortunate enough to see them in real life, you at least get to see them in this program. (Linus, 7)" | Edelbring et al., 2011 |
| 1. Goal | 1.8. Patient-centred care | Exposure to different types of patient personalities, cultural competency, interaction with patient | "Glimpses of the life world of the VP cases also stimulated forming ideas about patients’ personalities and their life situations. The names of the four cases were remembered at the time of the interview although students mostly referred to them by their respective diagnoses." | Edelbring et al., 2011 |
| 1. Goal | 1.9. Other | Other statements, not matching any other subtheme or too general to assign to a specific code | "Overall, it was a satisfying experience and time spent with VPs was believed to enhance the educational value of the clinical placement." | Edelbring et al., 2011 |
| 2. Phase | 2.1. Early stage | Pre-clinical years, basic sciences | "students of the pre- clinical years show a high preference in the adoption of VPs as learning activities. That could be explained from the lack of any clinical contact with real patients in their two first years of study and their willingness to have early, even virtual, clinical encounters." | Dafli et al., 2019 |
| 2. Phase | 2.2. Late stage | Clinical years, clerkships | "At the same time, there is a decrease of interest for VPs’ curricular adoption in the next years of study, something that is reversed again in the last (6th) year of study. This is the time when medical students often desire to expand their clinical experience and practical skills acquisition, since they will soon be active members of the healthcare workforce." | Dafli et al., 2019 |
| 3. Resources | 3.1. Limitations' awareness | Awareness about the constraints of introducing VPs in the curriculum | "In our study, the costs generated by the implementation process and by the maintenance of the application ranked low on everybody’s agenda; however, the leaders seemed more cost-conscious than other stakeholders." | Botezatu et al., 2010b |
| 3. Resources | 3.2. VPs exchange | Exchange and collaboration as a way to deal with limited resources | "Furthermore, the cross-cultural development and international exchange of VPs is also a very promising trend, which may help educational institutions all over the world to create extensive repositories of VPs that fit their local curriculum." | Fors et al., 2009 |
| 3. Resources | 3.3. Teachers' availability and effort | Dealing with a limited number of teachers competent in VPs and limited time they are able/willing to engage. | "Feasibility plays a major role when integrating e-learning into the curriculum. One needs to consider how many experienced instructors are available to manage the extra effort as well as the adequacy of the technical infrastructure at the faculty." | Hege et al., 2007 |
| 4. Alignment | 4.1. Local specificity | A need for adapting VPs to local context: language, cultural & socio-economic aspects, specific medical conditions | "However, as these may be only available in English, it is important to consider if VPs reflecting the local illness panorama and medical investigation procedures are needed." | Fors et al., 2009 |
| 4. Alignment | 4.2. Alignment strategies | A need to align or map VPs with elements of the curriculum (i.e. check the alignment between modalities (presence in assessment), ensure that the order in curriculum is correct (do not introduce VPs when some prerequisites were not addressed earlier) | "It would be nice if we were able to instantly recognise which educational sessions are connected, including lectures and sessions with real patients." | Huwendiek et al., 2013 |
| 4. Alignment | 4.3. Learning objectives | Identification of LO's which could be targeted with VPs | "Examples of learning outcomes that could be targeted using VPs include: Understanding global health issues and possibilities/ challenges in different parts of the world..." | Fors et al., 2009 |
| 4. Alignment | 4.4. VP selection criteria | Strategies for selection of VPs such as: selection of the number of VPs, difficulty level, navigation model, needs of particular medical discipline (e.g. internal medicine, paediatrics), prevalence of the depicted disease. | "The students voiced for numerous cases within a sub-speciality, with a minimum of 2–3 cases per disease. The professors also highlighted the importance of a ‘‘critical mass’’ of cases, but underlined that creating numerous cases is time-consuming." | Botezatu et al., 2010b |
| 5. Prioritisation | 5.1. Mandatory strategy | Obligation to complete a set number of VPs in a fixed time to get credit from/pass a course | "I think it makes sense to make at least the first VP compulsory, as this would allow every student to get acquainted with the programme and work with it. Afterwards they can decide for themselves whether they consider the VPs helpful or not." | Huwendiek et al., 2013 |
| 5. Prioritisation | 5.2. Exam-relevance | Clear statement that a specified part of the VP collection will be checked at the final examination | "Naturally, when I knew the contents of VP cases were likely to come up in the exam, I went over the cases a second time. I wrote out explicitly what the cardinal symptoms were, what the typical differential diagnoses were, what diagnostic investigations I ought to perform, what symptoms I needed to watch out for and how to plan the treatment. This is how I made my notes for every VP." | Huwendiek et al., 2013 |
| 5. Prioritisation | 5.3. Processing intensity | High engagement of teachers who intensively use, refer to in other activities, give feedback to the use of VPs. Combined, complex use. | "The main findings show that perceived benefit of VPs (PB) and log-in proportions were higher in settings with high case processing intensity." | Edelbring et al., 2012 |
| 5. Prioritisation | 5.4. Adaptation | Adjustment of VPs to local needs, culture | "Moreover, students and other users often feel that the degree of realism, and thus the engagement, is improved when the VPs reflect what really can be seen in society, with all its diversity. For example, the students at El Bosque in Colombia mentioned that because their cases ‘‘spoke’’ as locals and reflected the local conditions (and not the European or Anglo- American context), their engagement and motivation increased." | Fors et al., 2009 |
| 5. Prioritisation | 5.5. Visibility | Promotion of VPs in description of courses, syllabi, curriculum; Comprehensive description of their use, benefits. | "Knowing how VPs were linked to other activities motivated students to take self-study more seriously and consequently improved their learning." | Huwendiek et al., 2013 |
| 5. Prioritisation | 5.6. Authenticity | Perceived congruence of the VP with clinical practice. Evidence, testimonials of similarity of presented scenarios to practise. Realism of the VPs. Possibility to follow the case to the end - case closure. | "Our findings suggest that student engagement with VPs is not simply based on the quality of the VP resource but how these are perceived as beneficial to the student in relation to both educational and clinical attainment." | McCarthy et al., 2015 |
| 5. Prioritisation | 5.7. Peer opinion | Opinions about VPs circulating among students and teachers (e.g., last year's students; those who passed the exams; etc.). Hidden curriculum. | "The centrality of peer influence in creating a culture of use and promoting integration of the VPs into the student learning experience." | McCarthy et al., 2015 |
| 5. Prioritisation | 5.8. Time availability | Securing enough time for the use of VPs; VPs are not competing with other activities (like real patient exposure); Elimination of other activities to make space for VPs | "They sometimes had to face the dilemma of prioritising between VP study and patient work at the ward, clinical time being precious." | Edelbring, 2011 |
| 6. Relation | 6.1. General | General and non-specific statements regarding positioning VPs in relation to other forms of teaching. Includes comments on using VPs as replacement and supplement to other (non-specific) forms of learning. | "We found a trend toward more successful perceived implementation if they had either replaced or integrated VPs into an existing learning activity, as opposed to adding VPs on top of their existing activities." | Lang et al., 2013 |
| 6. Relation | 6.2. Expository methods | Association of VPs with expository educational methods like lectures, text-books or on-line readings. VPs help to apply the knowledge from lecture, and make the content presented in lectures more relevant; VPs allow relaxed referral to textbooks, not possible at bedside; more interactive than textbooks | "In a VP task, students can apply knowledge from the preceding lecture." | Huwendiek et al., 2013 |
| 6. Relation | 6.3. Simulations | i.e. SPs, skills lab, simulation - extends SP by the possibility of realistic anatomical representation of pathologies; VP not enough for physical examination and use of equipment | "The ability to present an unusual and important physical abnormality to a student couched in the context of a standardized patient delivering the history. Before NERVE, there was really no way to bring those two components together – the realistic patient-physician interactions that are possible with standardized patients, and the correct interactive anatomic representations of CN palsies that could not exist without the use of simulations." | Hirumi et al., 2016 |
| 6. Relation | 6.4. Clinical teaching | i.e. bedside teaching, clinical clerkships - VPs should not replace or compete with this form of education; VPs give an opportunity to reflect on the patient encounter as meeting RP is connected with emotions; VPs are more structured and relaxed than meeting RP. | "Therese: And that you don’t have to be so... when we meet patients, we’re really focused, sometimes it’s almost like having a memory lapse afterwards because you’ve been so incredibly focused during the visit. And here you didn’t need to be as focused; instead, you could really accept not being a full fledged doctor, you don’t know everything and so on. (Therese and Lotten, 5)" | Edelbring et al., 2011 |
| 6. Relation | 6.5. Small groups | i.e. PBL, follow-up seminars - working in small groups allows asking questions, clarifying difficulties, but should not be used to repeat the content of VPs as this is perceived as boring. Follow-up seminars motivate to a more thorough study of VPs | "Face-to-face discussion sessions should not revisit the preceding VPs but use methods to foster clinical reasoning in an interactive small group setting." | Huwendiek et al., 2013 |
| 6. Relation | 6.6. Optimal sequence | Recommended order of using VPs in relation to other forms of teaching | "So the order should be: lecture, VP, small group discussion, real patient and the contents of the sessions should match.’’ | Huwendiek et al., 2013 |
| 6. Relation | 6.7. Free choice of learning methods | Value diversity in learning styles, students prefer to make their own selection of modality how to study | "Many of us have alternative and varied resources that we are used to using for the past 2 years, so we did not like being forced to use a new resource that we felt we didn’t learn as effectively from. We believe that this should be a supplementary tool, not a mandatory part of the curriculum because we learn different ways." | Hirumi et al., 2016 |
| 7. Activities | 7.1. Questions | Asking questions related to the content of the VP | "address the embedded questions as well as guiding questions at the end of the case." | Kulasegaram et al., 2018 |
| 7. Activities | 7.2. Case presentations | Describing/explaining/presenting the case to the other students or to the teacher; documentation of a case in a standard/institutional form | "It really helps me when we go over the case again in a structured manner during the small group session. The tutors expected us to present the VPs to the group." | Huwendiek et al., 2013 |
| 7. Activities | 7.3. Book referral | Consulting a book while solving a case. | "But here you need to reflect more, you need to look up things in books more. (Roger, 13)" | Edelbring et al., 2011 |
| 7. Activities | 7.4. Similar case comparisons | Comparing and contrasting two or more similar cases to recognise key diagnostic features | "Wrap-up discussions in which two VPs with relevant differential diagnoses were compared improved students’ understanding of disease and helped them recognize key diagnostic features." | Huwendiek et al., 2013 |
| 7. Activities | 7.5. Checklists | Using standard/institutional checklists while going through the case | "Students used a comprehensive health his- tory form and a skills checklist form to conduct the history and physical examination with the virtual patient. These were the same forms used to practice skills in the in-person laboratory." | Kelley et al., 2015 |
| 7. Activities | 7.6. Lack of activities | Missing activities around VPs | "The lack of teacher control surprised the students; in particular they had expected to discuss cases with clinical teachers." | Edelbring et al., 2011 |
| 8. Time | 8.1. Time allocation | Regulation of time spent with VPs, fixed allocated time slots to interact with VPs | "Students appreciated dedicated time for self-study with VPs." | Huwendiek et al., 2013 |
| 8. Time | 8.2. Time efficiency | Interaction with more VPs in the same time than real patients | "It was also more time-efficient than interacting with real patients or participating in case seminars." | Edelbring et al., 2011 |
| 8. Time | 8.3. Deadlines | Setting up fixed deadlines, as external motivation | "I would have preferred a set time to do it and to sit a couple of hours so it doesn’t turn into rushing through a case. (Veronica, 6)" | Edelbring et al., 2011 |
| 9. Group | 9.1. Group assignment | Different perceptions as working on VPs in groups | "You get so much more from the situation when you discuss things with someone else, than if you would be working alone. (Camilla, 13)" | Edelbring et al., 2011 |
| 9. Group | 9.2. Individual assignments | Different perceptions as working on VPs individually | "A large majority (79%) reported having worked individually with the VPs, but only 56% answered that they preferred the individual study setting." | Edelbring et al., 2012 |
| 9. Group | 9.3. Group size | Statements about working with VPs in small vs big groups, where small groups take preference | "Smaller group sizes (such as in settings C and D) increase the exposure of and impose greater responsibility on each student at the seminar. It is likely that these students put more effort into processing the VPs before the follow-up occasion and were more alert during the seminar." | Edelbring et al., 2012 |
| 10. Presence | 10.1. Face-2-face | Statements about working with VPs in a face-2-face setting. Some components of VP activities need to be done face-2-face. | "The laboratory requirement for Web-based students is met with an intensive in-person laboratory, which meets on consecutive days during the semester." | Kelley et al., 2015 |
| 10. Presence | 10.2. Online learning | Statements about working at home or other location outside university | ". . . if you can work through a VP at home, you can check your knowledge about a certain topic by working through the relevant VP to see how you would do in a more realistic situation." (1,6) | Huwendiek et al., 2013 |
| 10. Presence | 10.3. Blended learning | Combination of two modes | "Blended activities can be used to achieve almost any educational objective, with the virtual patient acting as one component among many. We have used this approach for undergraduate, postgraduate and continuing professional development purposes. We have also successfully used virtual patients as the integrating or scaffolding medium for distributed teaching, where different groups at different sites work independently, and then come together for collaborative virtual patient tasks." | Ellaway et al., 2015 |
| 11. Orientation | 11.1. Faculty Development | Orienting teachers regarding required knowledge and skills include: awareness of the VP platform, VP cases, role in the curriculum, but also small group facilitation. Includes also methodological support from a community of teachers. | "Students considered it important for teachers to be well informed about the substance and curricular integration of the VPs. They also expected teachers to be experienced clinicians and have skills to facilitate small group discussions on clinical reasoning, for example contrasting different cases, quizzing students about confirming and disconfirming features of differential diagnoses and highlighting relevant clinical aspects. The learning gains from tutor-led wrap-up sessions depended strongly on the tutor’s facilitating skills and clinical knowledge." | Huwendiek et al., 2013 |
| 11. Orientation | 11.2. Students | Orienting students regarding required knowledge and skills include: the role of VPs in the curriculum (its relevance), schedule, language skills in the language of the VP. | "Some respondents wanted more instructions beforehand concerning VP work and the seminar discussions." | Edelbring et al., 2012 |
| 12. Infrastructure | 12.1. Stable Internet | Connecting with internet with no interruptions | "We observed some degree of consensus on the necessity of having a functional system (mainly as internet connection, logins, IT support);" | Botezatu et al., 2010b |
| 12. Infrastructure | 12.2. Security | Data protected by a login mechanism | "Our entire team had some technical difficulties, whether during the log-in process or during the patient interviews themselves and felt that our learning was somewhat compromised by this." | Hirumi et al., 2016 |
| 12. Infrastructure | 12.3. Usability | User-friendly VP interface | "The software should be easy-to-use, highly accessible" | Hege et al., 2007 |
| 12. Infrastructure | 12.4. Interoperability | Support for standard of exchanging VPs among universities and access to an IT helpdesk. | "The option to acquire VPs from other universities may, therefore, be appealing. Emerging standards for international cross-system exchange of VPs will facilitate this." | Fors et al., 2009 |
| 12. Infrastructure | 12.5. Robust software | VP software well tested for errors and able to handle high load of users | "Technical issues were identified during expert reviews, one-to-one and small group evaluations, and repeated tests by all members of the R&D team. We also implemented a code freeze, conducted a load test, and focused on debugging the system a week prior to the field-test, but evidently, such efforts were not sufficient." | Hirumi et al., 2016 |
| 12. Infrastructure | 12.6. IT support | A helpdesk for IT support in case of technical problems | "User support and case maintenance are essential." | Hege et al., 2007 |
| 13. Quality & Sustainability | 13.1. Extension of existing VP collections | Continuous creation of new cases. need to add new cases to the collection | "They were united though in considering that the continuous creation of new cases is essential to the post-implementation setting." | Botezatu et al., 2010b |
| 13. Quality & Sustainability | 13.2. VP review & update | VP quality control | "Because local conditions and regulatory requirements change over time, we recommend that clerkship directors periodically reevaluate their goals and objectives for VPs and other instructional activities." | Lang et al., 2013 |
| 13. Quality & Sustainability | 13.3. Student evaluation | Questionnaires, feedback session and ethnographic approaches | "These evaluations have focused on learning processes and how they align with intended outcomes. For example, we are using ethnographic approaches to evaluate how students are interacting with VPs in small groups." | Kulasegaram et al., 2018 |
| 14. Assessment | 14.1. Summative assessment | Use of VPs for decision of course credit, mastering a competence, selection of the best students | "The virtual patient assignments accounted for 17.5% of the total course grade. Focused written examinations, focused oral presentations, a final head-to-toe assessment, and documentation on a live patient and a multiple-choice examination accounted for the majority of the class grade." | Kelley et al., 2015 |
| 14. Assessment | 14.2. Feedback | Value of feedback, the forms or content of feedback that can be given to students in response to their performance | "Making concluding remarks about the case, highlighting what was especially important about it and getting helpful feedback on our performance has definitely helped me to enjoy a more fruitful learning experience.’’ (2,4)" | Huwendiek et al., 2013 |
| 14. Assessment | 14.3. Formative assessment | Monitoring of student's learning, identification of their strengths and weaknesses and target areas for improvement. | "The students consider VPS to be a more didactic form of evaluation and an intrinsically better evaluation tool than traditional exams ”VPS evaluation lets you see your strengths and weaknesses, where you are failing and what you need to improve, while on a paper exam one can, many times, get it right just by chance“ (student 15)." | Botezatu et al., 2010a |
| 14. Assessment | 14.4. Learning analytics | Methods for making conclusions from detailed digital records of students actions in the VP environment | "Evidently, formative and summative feedback received from erroneous interactions with the VPs may reveal misconceptions about CN pathology and push students to engage in ways that are not necessarily measured by embedded performance tests." | Hirumi et al., 2016 |
