# Supplementary material for: Integrating virtual patients into undergraduate health professions curricula: a framework synthesis of stakeholders’ opinions based on a systematic literature review
Source: BMC Med Educ. 2024 Jul 5;24:727. doi: 10.1186/s12909-024-05719-1 (PMC11225252; doi:10.1186/s12909-024-05719-1)
Supplement: Supplementary file 3 — Supplementary Material 3 [file 12909_2024_5719_MOESM3_ESM.docx]

**Additional file 3: Quality assessment of included studies using the QuADS tool**

| **Study** | **#1** | **#2** | **#3** | **#4** | **#5** | **#6** | **#7** | **#8** | **#9** | **#10** | **#11** | **#12** | **#13** | **Score** | **%** |
| --- | --- | --- | --- | --- | --- | --- | --- | --- | --- | --- | --- | --- | --- | --- | --- |
| Berman et al., 2009 | 1 | 2 | 3 | 3 | 3 | 3 | 3 | 3 | 3 | 3 | 3 | 2 | 3 | 35 | 90% |
| Botezatu et al., 2010a | 0 | 3 | 3 | 3 | 2 | 3 | 2 | 3 | 3 | 2 | 3 | 0 | 1 | 28 | 72% |
| Botezatu et al., 2010b | 1 | 2 | 3 | 3 | 1 | 1 | 2 | 0 | 2 | 3 | 2 | 0 | 1 | 21 | 54% |
| Dafli et al., 2019 | 1 | 3 | 3 | 2 | 1 | 1 | 1 | 1 | 2 | 3 | 3 | 1 | 1 | 23 | 59% |
| Dahri et al., 2019 | 1 | 3 | 3 | 3 | 1 | 2 | 3 | 2 | 3 | 2 | 3 | 3 | 3 | 32 | 82% |
| Edelbring et al., 2011 | 3 | 3 | 3 | 3 | 1 | 1 | 2 | 3 | 3 | 3 | 3 | 0 | 3 | 31 | 79% |
| Edelbring et al., 2012 | 3 | 3 | 3 | 2 | 0 | 3 | 3 | 1 | 3 | 2 | 3 | 0 | 3 | 29 | 74% |
| Huwendiek et al., 2013 | 3 | 3 | 1 | 3 | 2 | 1 | 3 | 2 | 3 | 3 | 3 | 0 | 3 | 30 | 77% |
| Kassianos et al., 2023 | 3 | 3 | 2 | 3 | 3 | 3 | 1 | 3 | 2 | 2 | 3 | 1 | 3 | 32 | 82% |
| Lang et al., 2013 | 3 | 3 | 3 | 2 | 3 | 3 | 2 | 3 | 3 | 3 | 3 | 2 | 3 | 36 | 92% |
| McCarthy et al., 2015 | 1 | 3 | 3 | 1 | 2 | 1 | 2 | 3 | 3 | 1 | 3 | 1 | 3 | 27 | 69% |
| Schifferdecker et al., 2012 | 3 | 3 | 3 | 3 | 3 | 3 | 3 | 3 | 3 | 2 | 3 | 2 | 2 | 36 | 92% |

**Legend:** QuADS scores 0: not reported; 1: reported, but inadequate, 2: reported and partially adequate; 3: sufficiently reported
